# Epigenetic control of effectors in plant pathogens

**DOI:** 10.3389/fpls.2014.00638

**Published:** 2014-11-12

**Authors:** Mark Gijzen, Chelsea Ishmael, Sirjana D. Shrestha

**Affiliations:** ^1^Agriculture and Agri-Food CanadaLondon, ON, Canada; ^2^Department of Biology, University of Western OntarioLondon, ON, Canada

**Keywords:** avirulence, detection, gene silencing, immunity, pathotype, virulence, R-gene, transposon

## Abstract

Plant pathogens display impressive versatility in adapting to host immune systems. Pathogen effector proteins facilitate disease but can become avirulence (Avr) factors when the host acquires discrete recognition capabilities that trigger immunity. The mechanisms that lead to changes to pathogen Avr factors that enable escape from host immunity are diverse, and include epigenetic switches that allow for reuse or recycling of effectors. This perspective outlines possibilities of how epigenetic control of *Avr* effector gene expression may have arisen and persisted in filamentous plant pathogens, and how it presents special problems for diagnosis and detection of specific pathogen strains or pathotypes.

## INTRODUCTION

In our material world, everyone faces the dilemma of what to keep and discard as priorities and needs change over time. It is an easy metaphor to compare the accumulation of possessions by a household to the accumulation of genes within a genome; however, the forces of natural selection are extemporaneous and cannot plan for the future. Nonetheless, the underlying molecular systems that continually edit a genome will themselves be affected by successes and failures that occur only after the passage of time. Genes and other genetic elements can be sorted, deleted, retained, and regulated within a genome in a way that maximizes survivorship in the future. This is the concept briefly explored here, using the example of effector proteins from filamentous plant pathogens that interact with host plant immune systems.

## WHY DO PATHOGENS HAVE Avr FACTORS?

Plant pathogen effector proteins represent finely tuned instruments of infection that are crucial to the parasitic lifestyle (Hogenhout et al., 2009; Rovenich et al., 2014). Effectors establish conditions that permit pathogenic organisms to grow and reproduce on their hosts. But an asset can become a liability when host plant immune systems evolve recognition capabilities that home in on effectors as triggers to activate robust defense responses that arrest pathogen growth (Dangl et al., 2013). In such cases of effector triggered immunity (ETI), the pathogen effector responsible for host immune activation is known as an avirulence (Avr) factor (de Wit, 2007). Pathogen Avr factors always raise simplistic questions when they are introduced to students of plant pathology. Why do pathogens possess Avr factors, since they are self-defeating? Why haven’t all the Avr factors been eliminated by natural selection? The answers of course hinge on the conditionality of ETI and on the utility of Avr factors in circumstances where they escape host immune recognition systems.

## GAIN OF VIRULENCE IS ESCAPE FROM IMMUNITY

When a pathogen effector comes under host immune surveillance, the corresponding *Avr* gene needs to change in some way, so that the Avr factor no longer causes ETI, in order for the pathogen to grow and reproduce on hosts with the enhanced immune capability. The necessary gain of virulence changes will depend on how the host immune system interacts with the effector (Stergiopoulos and de Wit, 2009). Conventional mutations to *Avr* genes that result in gain of virulence are exceedingly diverse (de Wit et al., 2009; Takken and Rep, 2010; Valent and Khang, 2010; Ravensdale et al., 2011; Jiang and Tyler, 2012; Huang et al., 2014; Vleeshouwers and Oliver, 2014). Pseudogenization due to interruption of the *Avr* open reading frame and wholesale *Avr* gene losses are common. This observation points to the dispensability of individual *Avr* genes and suggests that pathogen effector arsenals have a built-in redundancy. Provided that an Avr factor is retained by some individuals within a pathogen population, sub-populations or lineages can apparently lose and recover *Avr* genes repeatedly, as circumstances warrant. For example, in an asexually recombining population of a fungal pathogen, deleted *Avr* genes can be regained from other members of the same population (Chuma et al., 2011). There are also numerous examples of gain of virulence changes to pathogen *Avr* genes that are accomplished without any nucleotide changes whatsoever occurring within the open reading frame (Kang et al., 2001; Shan et al., 2004; Qutob et al., 2009; Dou et al., 2010; Dong et al., 2011; Gilroy et al., 2011; Qutob et al., 2013; Ali et al., 2014; Na et al., 2014). This can occur by epiallelic variation or by conventional mutations to *cis*-elements or flanking sequences that provide regulatory control of *Avr* gene transcription or translation, or possibly by changes to trans-acting elements, such as transcription- or epigenetic-factors that control *Avr* gene expression.

## NATURALLY OCCURRING REVERSIBLE SYSTEMS FOR *Avr* EXPRESSION STATES

**FIGURE 1:**
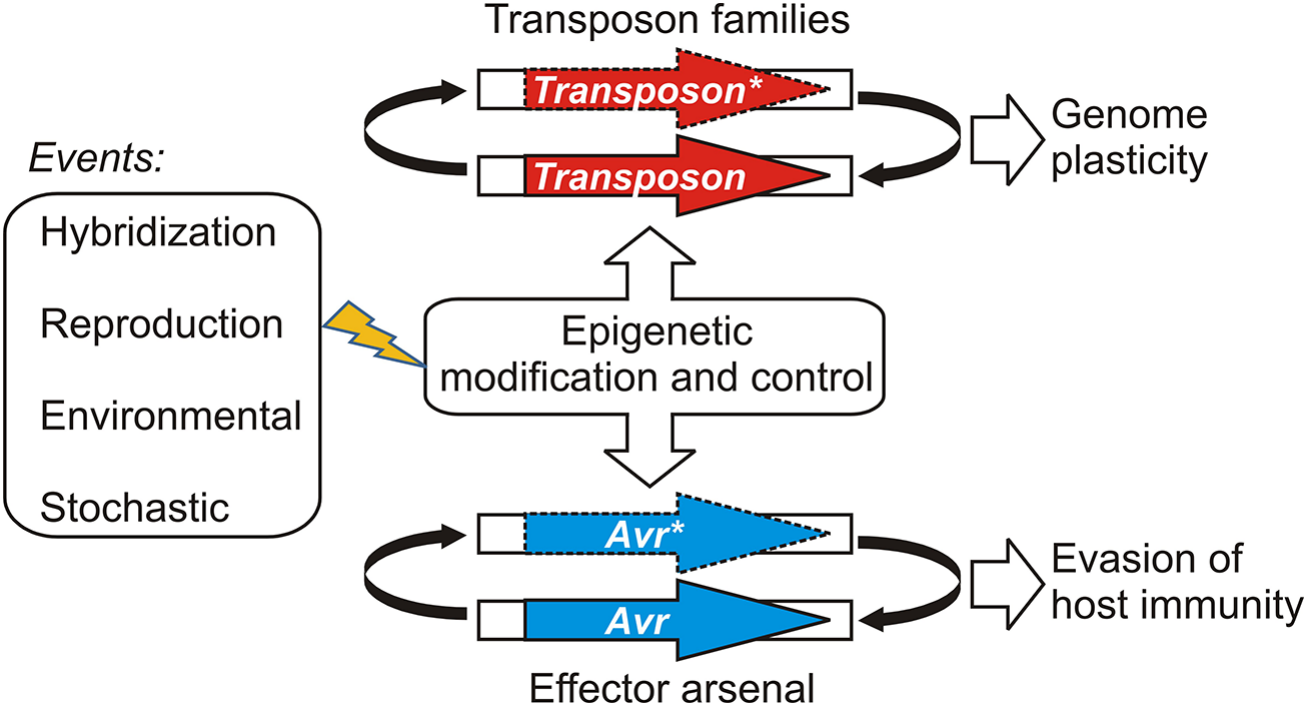
**A model illustrating how epigenetic control systems dedicated to transposon regulation may be shared or co-opted by pathogen effectors.** Events that have the potential to perturb the epigenetic stasis could alter transposon activity or avirulence (*Avr*) gene expression, leading to changes in genome organization or virulence, respectively. Active transposons or expressed *Avr* effectors are shown as bold arrows; silenced transposons and *Avr* effectors are shown with dashed lines and an asterisk (*).

Gain of virulence changes that leave the open reading frame of the *Avr* gene itself unchanged may be more easily reversible, should appropriate conditions occur for the re-deployment of an *Avr* allele that has been suppressed or wiped out of the population through a selective sweep. Epigenetic systems that regulate transcription offer powerful and versatile means for reversibly controlling effector gene expression states depending on environmental conditions. For example, experiments suggest that histone H3 lysine 9 (H3K9) methylation plays an role in coordinating the expression of hundreds of effectors in a fungal plant pathogen (Soyer et al., 2014). Epigenetic control of effector genes could arise in various ways, such as by recruiting existing systems that regulate the transcription and proliferation of mobile genetic elements or transposons (Sacristan et al., 2009; Kasuga et al., 2012; Whisson et al., 2012). This model is illustrated in **Figure 1**. Ancient epigenetic systems that release and silence transposons are proposed to contribute to genome plasticity and to be vitally important to evolutionary adaptation (Fedoroff, 2012). Whole genome sequencing of filamentous plant pathogens has provided good examples demonstrating that effectors and other genes associated with pathogenicity, host-range, and virulence are often embedded in repetitive, transposon-rich segments (Tyler et al., 2006; Haas et al., 2009; Spanu et al., 2010). The positioning of effector genes in such regions is advantageous because it facilitates duplication, recombination, and mutational mechanisms that lead to variation and often underlie gain of virulence changes (Van de Wouw et al., 2010; Rouxel et al., 2011; Raffaele and Kamoun, 2012). The close association with transposons could have allowed effector genes to co-opt or capture epigenetic switches dedicated to regulating the mobile genetic elements (Vetukuri et al., 2013). There is also evidence indicating that epigenetic regulators themselves can be highly polymorphic and positioned in repetitive, transposon-rich segments, suggesting adaptive interplay between conventional- and epigenetic variations in plant pathogens (Raffaele et al., 2010).

**FIGURE 2:**
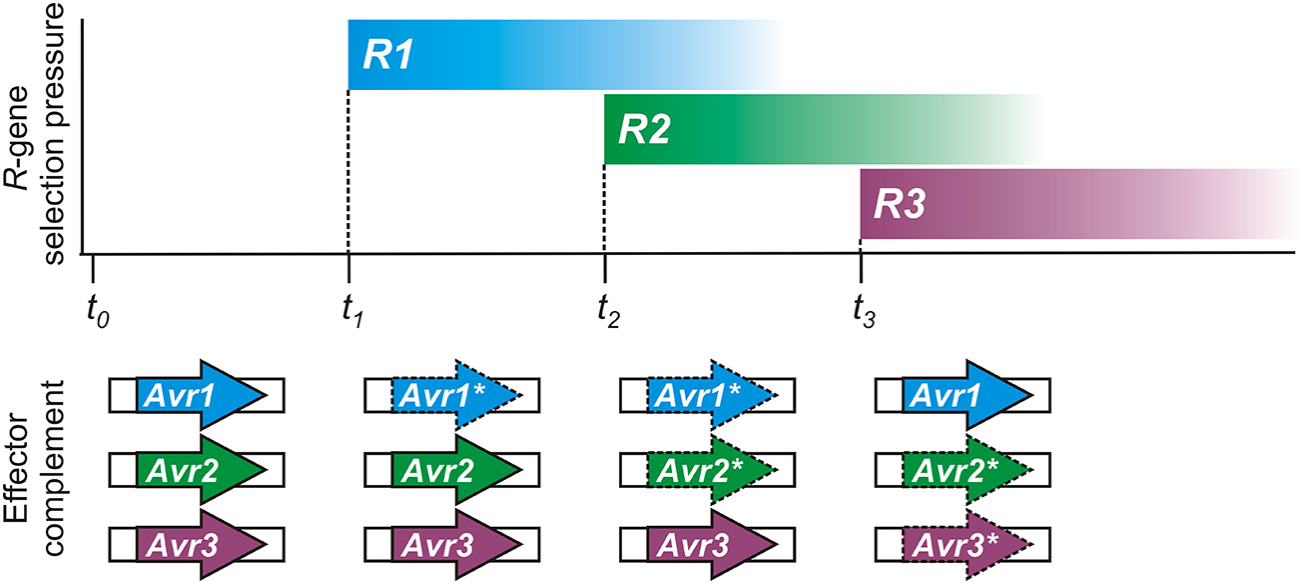
**A hypothetical timeline demonstrating how epigenetic regulation of pathogen avirulence (*Avr*) genes could respond to host resistance (*R*) gene selection pressure.** Reversible expression changes could result in recycling or reuse of *Avr* genes by pathogen populations, as shown in this illustration. Changes in plant *R*-gene selective pressure can result from genetic drift or cultivation practices. The complement of expressed effector genes, necessary to evade immunity with a minimum fitness penalty, is shown below for the pathogen. Expressed *Avr* effectors are shown as bold arrows; gene-silenced *Avr* effectors are shown with dashed lines and an asterisk (*).

Conceptually, the flexible nature of epigenetic control appears tailor-made for pathogen *Avr* effector genes, as illustrated in **Figure 2**. Spontaneous changes in *Avr* gene expression states have been experimentally observed (Na et al., 2014) and could help to account for previous descriptions of variation in virulence of clonal isolates in the laboratory (Rutherford et al., 1985) or in the field (Goodwin et al., 1995; Hovmøller and Justesen, 2007). Historically, plant pathologists have always struggled to explain changes in virulence of pathogen populations, and have noted that as specific gain of virulence changes accumulate in a pathogen strain, it loses general fitness or aggressiveness (Van der Plank, 1968; Watson, 1970).

Epigenetic switching of expression allows the pathogen to respond successfully to new immune capabilities of the host while retaining *Avr* genes for future use, if necessary. For example, host immunity in natural or cultivated plant populations may change as a result of genetic drift or agronomic practices. An immune receptor encoded by a host resistance (*R*) gene can be easily lost or mutated, or fall in to disuse, in the absence of any selective pressure or economic advantage, especially so if there is a fitness cost. The pathogen could also jump to a new host, or be introduced to a new environment, where the Avr factor does not face the challenge of host immune recognition. Under such circumstances, re-activating or recycling previous *Avr* effector genes could impart a fitness benefit that aids pathogen growth and reproduction.

## EPIALLELIC VARIATION OF *Avr* GENE EXPRESSION PRESENTS PRACTICAL PROBLEMS

Most troublesome to plant breeders is that epigenetic variation in *Avr* gene expression allows pathogens to adapt rapidly and recycle effectors after a selective sweep imposed by cultivar specific *R*-gene deployment. Epiallelic variation of *Avr* effector gene expression also presents diagnostic and detection problems for plant pathologists aiming to develop pathotype-specific tests for better tracking, management, and control of plant disease causing organisms. This is significant because molecular tools are currently driving advances in plant breeding and pathogen diagnostics, as breeders, pathologists, inspectors, and regulators increasingly rely on genetic information for their decision making. Sequence data can be helpful in characterizing pathogen strains from the field and predicting the risk of *R*-gene failure (Van de Wouw and Howlett, 2012) but genetic tests based on DNA sequences do not have the capability to detect expression differences. Testing a gene expression state is more technically demanding and requires greater skill, time, and resources; it is not a test that is routinely performed by diagnostic laboratories. Perhaps inexpensive and efficient detection systems will be developed and applied to this problem in the future, such as for RNA molecules, or DNA or protein modifications that could underlie epigenetic variation. Until this occurs it does not seem likely that simple tests will be available for pathotyping strains that vary epigenetically in *Avr* gene expression. Traditional pathotyping relies on isolation, culturing, and phenotyping pathogen strains by inoculation on a set of plant lines carrying different *R*-genes. This demands considerable time and expertise, and requires plants, growth facilities, and other materials dedicated to each disease causing organism. The result is that strain pathotyping is usually done only by research laboratories that are specialized towards a particular organism or are well-versed in the proper bioassay.

## CONCLUSION

This perspective introduces the concept of epigenetic variation of effector expression in plant pathogens. It is a newly discovered phenomenon that is still in the early stages of being characterized. Epigenetic variation contributes to phenotypic diversity and host adaptation and helps to explain how disease causing and invasive organisms rapidly adapt to changing host and environmental conditions (Kasuga and Gijzen, 2013). The prevalence of epigenetic variation, its mechanistic control, its relative importance to particular plant pathogen species, and its interplay with conventional genetic variation all require further work to define. Are the known and proposed examples of epigenetic variation within plant pathogens unusual and rare curiosities, or do they point to a more widespread biological phenomenon with evolutionary implications that have been long overlooked or undetected? Many scientists suspect the latter may be true, but it is a question that deserves a clear answer. There are potentially wide-ranging consequences affecting disease management and diagnostics that arise from epigenetic control of *Avr* genes and other host-range determinants. This should spur additional research in the area, which can only be helpful for controlling plant diseases that are damaging to crops, ornamental plants, and natural environments.

## Conflict of Interest Statement

The authors declare that the research was conducted in the absence of any commercial or financial relationships that could be construed as a potential conflict of interest.

## References

[B1] Ali S. Laurie J. D. Linning R. Cervantes-Chavez J. A. Gaudet D. Bakkeren G. (2014). An immunity-triggering effector from the Barley smut fungus Ustilago hordei resides in an Ustilaginaceae-specific cluster bearing signs of transposable element-assisted evolution. *PLoS Pathog.* 10:e1004223 10.1371/journal.ppat.1004223 PMC4081816 24992661

[B2] Chuma I. Isobe C. Hotta Y. Ibaragi K. Futamata N. Kusaba M. (2011). Multiple translocation of the AVR-Pita effector gene among chromosomes of the rice blast fungus *Magnaporthe oryzae* and related species. *PLoS Pathog* 7:e1002147 10.1371/journal.ppat.1002147 PMC3145791 21829350

[B3] Dangl J. L. Horvath D. M. Staskawicz B. J. (2013). Pivoting the plant immune system from dissection to deployment. *Science* 341 746–751 10.1126/science.1236011 23950531 PMC3869199

[B4] de Wit P. J. (2007). How plants recognize pathogens and defend themselves. *Cell Mol. Life Sci.* 64 2726–2732 10.1007/s00018-007-7284-7 17876517 PMC11136299

[B5] de Wit P. J. Mehrabi R. Van den Burg H. A. Stergiopoulos I. (2009). Fungal effector proteins: past, present and future. *Mol. Plant Pathol.* 10 735–747 10.1111/j.1364-3703.2009.00591.x 19849781 PMC6640362

[B6] Dong S. Yu D. Cui L. Qutob D. Tedman-Jones J. Kale S. D. (2011). Sequence variants of the *Phytophthora sojae* RXLR effector Avr3a/5 are differentially recognized by Rps3a and Rps5 in soybean. *PLoS ONE* 6:e20172 10.1371/journal.pone.0020172 PMC3136461 21779316

[B7] Dou D. Kale S. D. Liu T. Tang Q. Wang X. Arredondo F. D. (2010). Different domains of *Phytophthora sojae* effector Avr4/6 are recognized by soybean resistance genes Rps4 and Rps6. *Mol. Plant Microbe Interact.* 23 425–435 10.1094/MPMI-23-4-0425 20192830

[B8] Fedoroff N. V. (2012). Presidential address. Transposable elements, epigenetics, and genome evolution. *Science* 338 758–767 10.1126/science.338.6108.758 23145453

[B9] Gilroy E. M. Breen S. Whisson S. C. Squires J. Hein I. Kaczmarek M. (2011). Presence/absence, differential expression and sequence polymorphisms between PiAVR2 and PiAVR2-like in *Phytophthora infestans* determine virulence on R2 plants. *New Phytol.* 191 763–776 10.1111/j.1469-8137.2011.03736.x 21539575

[B10] Goodwin S. B. Sujkowski L. S. Fry W. E. (1995). Rapid evolution of pathogenicity within clonal lineages of the potato late blight disease fungus. *Phytopathology* 85 669–676 10.1094/Phyto-85-669

[B11] Haas B. J. Kamoun S. Zody M. C. Jiang R. H. Handsaker R. E. Cano L. M. (2009). Genome sequence and analysis of the Irish potato famine pathogen *Phytophthora infestans*. *Nature* 461 393–398 10.1038/nature08358 19741609

[B12] Hogenhout S. A. Van der Hoorn R. A. L. Terauchi R. Kamoun S. (2009). Emerging concepts in effector biology of plant-associated organisms. *Mol. Plant Microbe Interact.* 22 115–122 10.1094/MPMI-22-2-0115 19132864

[B13] Hovmøller M. S. Justesen A. F. (2007). Rates of evolution of avirulence phenotypes and DNA markers in a northwest European population of *Puccinia striiformis* f. *sp. tritici. Mol. Ecol.* 16 4637–4647 10.1111/j.1365-294X.2007.03513.x 17887968

[B14] Huang J. Si W. Deng Q. Li P. Yang S. (2014). Rapid evolution of *avirulence* genes in rice blast fungus *Magnaporthe oryzae*. *BMC Genet.* 15:45 10.1186/1471-2156-15-45 PMC4021558 24725999

[B15] Jiang R. H. Tyler B. M. (2012). “Mechanisms and evolution of virulence in oomycetes. *Annu. Rev. Phytopathol.* 50 295–318 10.1146/annurev-phyto-081211-172912 22920560

[B16] Kang S. Lebrun M. H. Farrall L. Valent B. (2001). Gain of virulence caused by insertion of a Pot3 transposon in a *Magnaporthe grisea* avirulence gene. *Mol. Plant Microbe Interact.* 14 671–674 10.1094/MPMI.2001.14.5.671 11332731

[B17] Kasuga T. Gijzen M. (2013). Epigenetics and the evolution of virulence. *Trends Microbiol.* 21 575–582 10.1016/j.tim.2013.09.003 24095304

[B18] Kasuga T. Kozanitas M. Bui M. Huberli D. Rizzo D. M. Garbelotto M. (2012). Phenotypic diversification is associated with host-induced transposon derepression in the sudden oak death pathogen *Phytophthora ramorum*. *PLoS ONE* 7:e34728 10.1371/journal.pone.0034728 PMC3329494 22529930

[B19] Na R. Yu D. Chapman B. P. Zhang Y. Kuflu K. Austin R. (2014). Genome re-sequencing and functional analysis places the *Phytophthora sojae* avirulence genes Avr1c and Avr1a in a tandem repeat at a single locus. *PLoS ONE* 9:e89738 10.1371/journal.pone.0089738 PMC3933651 24586999

[B20] Qutob D. Patrick Chapman B. Gijzen M. (2013). Transgenerational gene silencing causes gain of virulence in a plant pathogen. *Nat. Commun.* 4 1349 10.1038/ncomms2354 PMC3562452 23322037

[B21] Qutob D. Tedman-Jones J. Dong S. Kuflu K. Pham H. Wang Y. (2009). Copy number variation and transcriptional polymorphisms of *Phytophthora sojae* RXLR effector genes Avr1a and Avr3a. *PLoS ONE* 4:e5066 10.1371/journal.pone.0005066 PMC2661136 19343173

[B22] Raffaele S. Farrer R. A. Cano L. M. Studholme D. J. MacLean D. Thines M. (2010). Genome evolution following host jumps in the Irish potato famine pathogen lineage. *Science* 330 1540–1543 10.1126/science.1193070 21148391

[B23] Raffaele S. Kamoun S. (2012). Genome evolution in filamentous plant pathogens: why bigger can be better. *Nat. Rev. Microbiol.* 10 417–430 10.1038/nrmicro2790 22565130

[B24] Ravensdale M. Nemri A. Thrall P. H. Ellis J. G. Dodds P. N. (2011). Co-evolutionary interactions between host resistance and pathogen effector genes in flax rust disease. *Mol. Plant Pathol.* 12 93–102 10.1111/j.1364-3703.2010.00657.x 21118351 PMC2999005

[B25] Rouxel T. Grandaubert J. Hane J. K. Hoede C. van de Wouw A. P. Couloux A. (2011). Effector diversification within compartments of the *Leptosphaeria maculans* genome affected by Repeat-Induced Point mutations. *Nat. Commun.* 2 202 10.1038/ncomms1189 PMC3105345 21326234

[B26] Rovenich H. Boshoven J. C. Thomma B. P. (2014). Filamentous pathogen effector functions: of pathogens, hosts and microbiomes. *Curr. Opin. Plant Biol.* 20C, 96–103 10.1016/j.pbi.2014.05.001 24879450

[B27] Rutherford F. S. Ward E. W. B. Buzzell R. I. (1985). Variation in virulence in successive single-zoospore propagations of *Phytophthora megasperma* f.sp.glycinea. *Phytopathology* 75 371–374 10.1094/Phyto-75-371

[B28] Sacristan S. Vigouroux M. Pedersen C. Skamnioti P. Thordal-Christensen H. Micali C. (2009). Coevolution between a family of parasite virulence effectors and a class of LINE-1 retrotransposons. *PLoS ONE* 4:e7463 10.1371/journal.pone.0007463 PMC2759079 19829700

[B29] Shan W. X. Cao M. Dan L. U. Tyler B. M. (2004). The Avr1b locus of *Phytophthora sojae* encodes an elicitor and a regulator required for avirulence on soybean plants carrying resistance gene Rps1b. *Mol. Plant Microbe Interact.* 17 394–403 10.1094/MPMI.2004.17.4.394 15077672

[B30] Soyer J. L. El Ghalid M. Glaser N. Ollivier B. Linglin J. Grandaubert J. (2014). Epigenetic control of effector gene expression in the plant pathogenic fungus Leptosphaeria maculans. *PLoS Genet.* 10:e1004227 10.1371/journal.pgen.1004227 PMC3945186 24603691

[B31] Spanu P. D. Abbott J. C. Amselem J. Burgis T. A. Soanes D. M. Stuber K. (2010). Genome expansion and gene loss in powdery mildew fungi reveal tradeoffs in extreme parasitism. *Science* 330 1543–1546 10.1126/science.1194573 21148392

[B32] Stergiopoulos I. de Wit P. J. (2009). Fungal effector proteins. *Annu. Rev. Phytopathol.* 47 233–263 10.1146/annurev.phyto.112408.132637 19400631

[B33] Takken F. Rep M. (2010). The arms race between tomato and *Fusarium oxysporum*. *Mol. Plant Pathol.* 11 309–314 10.1111/j.1364-3703.2009.00605.x 20447279 PMC6640361

[B34] Tyler B. M. Tripathy S. Zhang X. M. Dehal P. Jiang R. H. Y. Aerts A. (2006). Phytophthora genome sequences uncover evolutionary origins and mechanisms of pathogenesis. *Science* 313 1261–1266 10.1126/science.1128796 16946064

[B35] Valent B. Khang C. H. (2010). Recent advances in rice blast effector research. *Curr. Opin. Plant Biol.* 13 434–441 10.1016/j.pbi.2010.04.012 20627803

[B36] Van de Wouw A. P. Cozijnsen A. J. Hane J. K. Brunner P. C. McDonald B. A. Oliver R. P. (2010). Evolution of linked avirulence effectors in *Leptosphaeria maculans* is affected by genomic environment and exposure to resistance genes in host plants. *PLoS Pathog.* 6:e1001180 10.1371/journal.ppat.1001180 PMC2973834 21079787

[B37] Van de Wouw A. P. Howlett B. J. (2012). Estimating frequencies of virulent isolates in field populations of a plant pathogenic fungus, *Leptosphaeria maculans*, using high-throughput pyrosequencing. *J. Appl. Microbiol.* 113 1145–1153 10.1111/j.1365-2672.2012.05413.x 22830361

[B38] Van der Plank J. E. (1968). *Disease Resistance in Plants.* New York: Academic Press.

[B39] Vetukuri R. R. Asman A. K. Jahan S. N. Avrova A. O. Whisson S. C. Dixelius C. (2013). Phenotypic diversification by gene silencing in *Phytophthora* plant pathogens. *Commun. Integr. Biol.* 6:e25890 10.4161/cib.25890 PMC3917941 24563702

[B40] Vleeshouwers V. G. Oliver R. P. (2014). Effectors as tools in disease resistance breeding against biotrophic, hemibiotrophic, and necrotrophic plant pathogens. *Mol. Plant Microbe Interact.* 27 196–206 10.1094/MPMI-10-13-0313-IA 24405032

[B41] Watson I. A. (1970). Changes in virulence and population shifts in plant pathogens. *Annu. Rev. Phytopathol.* 8 209–230 10.1146/annurev.py.08.090170.001233

[B42] Whisson S. Vetukuri R. Avrova A. Dixelius C. (2012). Can silencing of transposons contribute to variation in effector gene expression in *Phytophthora infestans*? *Mob. Genet. Elements* 2 110–114 10.4161/mge.20265 22934246 PMC3429519

